# Q Fever in Migrant Workers, Scotland

**DOI:** 10.3201/eid1312.071058

**Published:** 2007-12

**Authors:** Kevin G.J. Pollock, Dominic J. Mellor, Lynda M. Browning, Louise Wilson, Martin Donaghy

**Affiliations:** * Health Protection Scotland, Glasgow, Scotland

**Keywords:** Coxiella burnetii, Q fever, migrant workers, letter

**To the Editor:** Q fever is a zoonosis caused by infection with *Coxiella burnetii* and is most commonly associated with occupational exposure to animal-slaughtering facilities. *C. burnetii* is an obligate intracellular bacterium and causes highly variable disease, ranging from asymptomatic infection to fatal chronic infective endocarditis. In June 2006, the United Kingdom experienced its largest outbreak of Q fever with 138 cases associated with a slaughterhouse near Stirling in Scotland. The slaughterhouse had been processing post-parturition ewes in the lairage (place for keeping livestock temporarily) at the end of May. These animals were thought to be among the most likely to shed the organism (*1*). Further investigation showed that a ewe had aborted in the lairage toward the end of May. Although the sheep lairage was the most likely source of the infection, no microbiologic evidence confirmed this, as *C. burnetii* was not isolated from environmental samples.

The outbreak was neither remarkable for its putative mode of transmission nor for the industry involved, but both the number and nationalities of migrant workers infected was noteworthy. Since 2004, 12 member states have joined the European Union and this has led to an influx of immigrants to the United Kingdom. The increase in migrant numbers has partly been a result of the government’s managed migration policy, expanding migration to fill vacancies in skilled and low-wage occupations. Employers have difficulty recruiting UK workers because of the jobs’ physical demands, long hours that limit social activities, and low pay. They therefore recruit international workers with a good work ethic and reliability; central and Eastern European workers are compared favorably with UK nationals (*2*). Migrants from Eastern and central Europe are now more likely to be found in low-wage occupations in agriculture, construction, hospitality, and au pair employment. Of the 138 cases of Q fever, 48 were immigrants from the following countries: Slovakia (41), Poland (3), Czech Republic (2), and Lithuania (2). Unsurprisingly, epidemiologic case interviews were beset with linguistic and logistic problems.

The diagnosis of Q fever relies predominantly on its serologic legacy since asymptomatic seroconversion occurs in up to 60% of patients (*3*). Analysis of our cohort found that non-UK patients were significantly less likely than their UK counterparts to have symptoms (fever, muscle pain, joint pain, headache, and cough) and to subsequently have Q fever confirmed (Table, p<0.001). Twenty-two patients (15 UK, 7 non-UK) did not complete epidemiologic questionnaires and were therefore not included in this analysis.

**Table:** χ^2^ analysis of Q fever symptoms and GP registration by nationality*

Characteristic	No. (%) UK natives		All
	Yes	No	
Symptoms			
No	19 (28.4)	25 (15.6)	44
Yes	56 (46.6)	16 (25.4)	72
All	75	41	116
GP registered			
No	1 (21.3)	32 (11.7)	33
Yes	77 (56.7)	11 (31.3)	88
All	78	43	121

*Expected nos. in parentheses. GP, general practitioner; UK, United Kingdom.

Furthermore, analysis of cases registered with general practitioners (GPs) identified a significant difference (Table, p<0.001) between UK and non-UK patients with the latter group less likely to be registered with a GP. Although most UK residents were registered with a general practice, only 11 of 43 non-UK cases were registered. Information on GP registration was not known for 17 patients, and these were not included in the analysis.

Although the investigating health board took stringent steps to ensure follow-up of all patients, we believe that some asymptomatic non-UK patients may have permanently returned to their native countries with undiagnosed illness, and subsequently, cannot be traced. This unfortunate scenario has potentially catastrophic implications for these patients because proper follow-up clinical management of Q fever is necessary to prevent possible endocarditis (*4*), unnecessary surgery, and premature death.

Persons with known occupational hazards have benefited from an effective Q fever vaccine; abattoir workers and farmers are routinely vaccinated in Australia (*5*). Given the aforementioned linguistic and coordination issues with follow-up of migrant workers and the potential gravity of inappropriate clinical follow-up, it may be prudent to consider Q fever vaccination for all employees who work within UK meat-processing industries.

Public health practitioners should be aware of the continuously evolving multinational makeup of the local population and this should stimulate constant review of local translation services because census data seriously underrecognize the ethnic minority migrant worker population. Furthermore, many migrant workers are unsure of their rights to access primary and hospital care and the structure of healthcare is unfamiliar to many. GPs should consider zoonotic infections, such as Q fever, when patients with acute febrile illness report occupational livestock exposure, especially because migrant workers have become an important source of labor (sometimes preferred over domestic workers) in the agricultural workforce in the United Kingdom (*2*).
